# Comparison of “IN-REC-SUR-E” and LISA in preterm neonates with respiratory distress syndrome: a randomized controlled trial (IN-REC-LISA trial)

**DOI:** 10.1186/s13063-024-08240-4

**Published:** 2024-07-02

**Authors:** Giovanni Vento, Angela Paladini, C. Aurilia, S. Alkan Ozdemir, V. P. Carnielli, F. Cools, S. Costa, F. Cota, C. Dani, P. G. Davis, S. Fattore, C. Fè, N. Finer, F. P. Fusco, C. Gizzi, E. Herting, M. Jian, A. Lio, G. Lista, F. Mosca, S. Nobile, A. Perri, S. Picone, J. J. Pillow, G. Polglase, T. Pasciuto, R. Pastorino, M. Tana, D. Tingay, C. Tirone, A. H. van Kaam, M. L. Ventura, A. Aceti, M. Agosti, G. Alighieri, G. Ancora, V. Angileri, G. Ausanio, S. Aversa, E. Balestri, E. Baraldi, M. C. Barbini, C. Barone, R. Beghini, C. Bellan, A. Berardi, I. Bernardo, P. Betta, M. Binotti, B. Bizzarri, G. Borgarello, S. Borgione, A. Borrelli, R. Bottino, G. Bracaglia, I. Bresesti, I. Burattini, C. Cacace, F. Calzolari, M. F. Campagnoli, L. Capasso, M. Capozza, M. G. Capretti, J. Caravetta, C. Carbonara, V. Cardilli, M. Carta, F. Castoldi, A. Castronovo, E. Cavalleri, F. Cavigioli, S. Cecchi, V. Chierici, C. Cimino, F. Cocca, C. Cocca, P. Cogo, M. Coma, V. Comito, V. Condò, C. Consigli, R. Conti, M. Corradi, G. Corsello, L. T. Corvaglia, A. Costa, A. Coscia, F. Cresi, F. Crispino, P. D’Amico, L. De Cosmo, C. De Maio, G. Del Campo, S. Di Credico, S. Di Fabio, P. Di Nicola, A. Di Paolo, S. Di Valerio, A. Distilo, V. Duca, A. Falcone, R. Falsaperla, V. A. Fasolato, V. Fatuzzo, F. Favini, M. P. Ferrarello, S. Ferrari, F. Fiori Nastro, C. A. Forcellini, A. Fracchiolla, A. Gabriele, F. Galdo, F. Gallini, A. Gangemi, G. Gargano, D. Gazzolo, M. P. Gentile, S. Ghirardello, F. Giardina, L. Giordano, E. Gitto, M. Giuffrè, L. Grappone, F. Grasso, I. Greco, A. Grison, R. Guglielmino, I. Guidotti, I. Guzzo, N. La Forgia, S. La Placa, G. La Torre, P. Lago, L. Lanciotti, A. Lavizzari, F. Leo, V. Leonardi, D. Lestingi, J. Li, P. Liberatore, D. Lodin, R. Lubrano, M. Lucente, S. Luciani, D. Luvarà, G. Maffei, A. Maggio, L. Maggio, K. Maiolo, L. Malaigia, G. Mangili, A. Manna, E. Maranella, A. Marciano, P. Marcozzi, M. Marletta, L. Marseglia, D. Martinelli, S. Martinelli, S. Massari, L. Massenzi, F. Matina, L. Mattia, G. Mescoli, I. V. Migliore, D. Minghetti, I. Mondello, S. Montano, G. Morandi, N. Mores, S. Morreale, I. Morselli, M. Motta, M. Napolitano, D. Nardo, A. Nicolardi, S. Nider, G. Nigro, M. Nuccio, L. Orfeo, C. Ottaviano, P. Paganin, S. Palamides, S. Palatta, P. Paolillo, M. G. Pappalardo, E. Pasta, L. Patti, G. Paviotti, R. Perniola, G. Perotti, S. Perrone, F. Petrillo, M. S. Piazza, A. Piccirillo, M. Pierro, E. Piga, G. A. Pingitore, S. Pisu, C. Pittini, F. Pontiggia, G. Pontrelli, A. Primavera, A. Proto, L. Quartulli, F. Raimondi, L. Ramenghi, M. Rapsomaniki, A. Ricotti, C. Rigotti, M. Rinaldi, F. M. Risso, E. Roma, E. Romanini, V. Romano, E. Rosati, V. Rosella, I. Rulli, V. Salvo, C. Sanfilippo, A. Sannia, A. Saporito, A. Sauna, E. Scapillati, F. Schettini, A. Scorrano, S. Semeria Mantelli, V. Sepporta, P. Sindico, A. Solinas, E. Sorrentino, E. Spaggiari, A. Staffler, M. Stella, D. Termini, G. Terrin, A. Testa, G. Tina, M. Tirantello, B. Tomasini, F. Tormena, L. Travan, D. Trevisanuto, G. Tuling, V. Tulino, L. Valenzano, S. Vedovato, S. Vendramin, P. E. Villani, S. Viola, V. Viola, G. Vitaliti, M. Vitaliti, P. Wanker, Y. Yang, S. Zanetta, E. Zannin

**Affiliations:** 1 Dipartimento Universitario Scienze Della Vita E Sanità Pubblica, UOC Di Neonatologia, Fondazione Policlinico Universitario Agostino Gemelli IRCCS - Università Cattolica del Sacro Cuore, Rome, Italy; 2 Health Science University Dr. Behcet Uz Children’s Hospital, Izmir, Turkey; 3 Azienda Ospedaliero Universitaria Ospedali Riuniti, Ancona, Italy; 4 Department of Neonatology, Universitair Ziekenhuis Brussel, Laarbeeklaan 101 - 1090, Brussel, Belgium; 5 Department of Mother and Child Health, Division of Neonatology and Neonatal Intensive Care Unit, Careggi University Hospital Florence, Firenze, Italy; 6 Neonatal Services, Royal Women’s Hospital, Melbourne, Australia; 7 School of Medicine, University of California, San Diego, San Diego, CA USA; 8 Unità operativa complessa di neonatologia e TIN Ospedale Sant’Eugenio, ASL ROMA 2, Rome, Italy; 9 Klinik für Kinder- und Jugendmedizin UNIVERSITÄTSKLINIKUM Schleswig-Holstein Campus Lübeck Ratzeburger Allee, 160 Haus A, Lübeck, 23538 Germany; 10 Shengjing Hospital of China Medical University, Shenyang, China; 11 Neonatologia e Terapia Intensiva Neonatale (TIN) Ospedale Dei Bambini “V.Buzzi” ASST-FBF-Sacco, Milan, 20154 Italy; 12 Department of Clinical Sciences and Community Health, University of Milan, Fondazione IRCCS Cà Granda Ospedale Maggiore Policlinico, Milan, Italy; 13 Policlinico Casilino, Rome, Italy; 14 Centre for Child Health Research and School of Human Sciences, The University of Western Australia, Perth, WA Australia; 15 The Ritchie Centre Hudson Institute of Medical Research and Department of Obstetrics and Gynaecology, Monash University, Clayton, Victoria 3168 Australia; 16 Research Core Facility Data Collection G-STeP, Fondazione Policlinico Universitario Agostino Gemelli IRCCS, Rome, 00168 Italy; 17 Section of Hygiene, University Department of Life Sciences and Public Health, Università Cattolica del Sacro Cuore, Rome, 00168 Italy; 18 Ospedale Maggiore, Bologna, Italy; 19 Fondazione IRCCS San Gerardo Dei Tintori, Monza, Italy; 20 Neonatal Research, Murdoch Children’s Research Institute, Melbourne, Australia; 21 Department of Neonatology, Emma Children’s Hospital, Amsterdam UMC, University of Amsterdam, Vrije Universiteit Amsterdam, Amsterdam, The Netherlands; 22 Policlinico S. Orsola-Malpighi, Università Di Bologna, Bologna, Italy; 23 Università Degli Studi Dell’Insubria, Varese, Italy; 24 P.P. “A. Perrino” Brindisi-ASL BR, Brindisi, Italy; 25 Ospedale Infermi, Rimini, Italy; 26 Presidio Ospedaliero Ingrassia ASP, Palermo, Italy; 27 Azienda Ospedaliera S.Anna-S.Sebastiano, Caserta, Italy; 28 Azienda Ospedaliera “Spedali Civili”, Brescia, Italy; 29 Arcispedale Santa Maria Nuova Reggio, Emilia, Italy; 30 University of Padua, Padua, Italy; 31 Ospedale Evangelico Betania, Naples, Italy; 32 Azienda Ospedaliera Bolognini, Seriate, BG Italy; 33 AOU Policlinico Modena, Modena, Italy; 34 Neonatologia Universitaria, Ospedale S.Anna – Città della Salute e della Scienza di Torino, Turin, Italy; 35 AOU Policlinico Vittorio Emanuele-Gaspare Rodolico Catania, Catania, Italy; 36 Azienda Ospedaliera S. Croce E Carle, Cuneo, Italy; 37 Azienda Ospedaliera Vito Fazzi, Lecce, Italy; 38 Bel Colle Hospital, Viterbo, Italy; 39 Barone Romeo Hospital, Patti, ME Italy; 40 Azienda OU, Parma, Italy; 41 Ospedale PO S. Anna, AOU Città Della Salute E Della Scienza, Turin, Italy; 42 Federico II University, Naples, Italy; 43 Policlinico Di Bari, Bari, Italy; 44 Policlinico Umberto I, Rome, Italy; 45 Università degli Studi, Palermo, Italy; 46 Azienda Ospedaliera Universitaria Senese, Siena, Italy; 47 Azienda OU Policlinico “Rodolico-San Marco”, Catania, Italy; 48 Ospedale San Pio, Benevento, Italy; 49 Azienda Sanitaria Universitaria Friuli Centrale, Udine, Italy; 50 AO Bianchi-Melacrino-Morelli Reggio Calabria, Reggio Calabria, Italy; 51 S.Pietro Fatebenefratelli, Rome, Italy; 52 Presidio Ospedaliero S. Antonio Abate – Azienda Sanitaria Provinciale, Trapani, Italy; 53 Di Venere Hospital, Bari, Italy; 54 Azienda Ospedaliera Per L’emergenza Cannizzaro, Catania, Italy; 55 U.O.C T.I.N. e Neonatologia, P.O.C Taranto “Santissima Annunziata”, Taranto, Italy; 56 San Salvatore Hospital, L’Aquila, Italy; 57 Azienda Ospedaliera San Giovanni Addolorata, Rome, Italy; 58 Azienda Ospedaliera Carlo Poma, Mantua, Italy; 59 ASST Papa Giovanni XXIII, Bergamo, Italy; 60 Azienda Ospedaliera-Universitaria Ospedali Riuniti Foggia, Foggia, Italy; 61 Maggiore Hospital, Novara, Italy; 62 Ospedale “Ss Annunziata”, Chieti - Università Degli Studi G. D’Annunzio Chieti, Pescara, Italy; 63 Azienza Ospedaliera Universitaria di Ferrara, Ferrara, Italy; 64 Università di Pavia, Pavia, Italy; 65 Ospedale Vincenzo Cervello, Palermo, Italy; 66 Pineta Grande, Castel Volturno, CE Italy; 67 Università degli Studi, Messina, Italy; 68 ARNAS Civico Hospital, Palermo, Italy; 69 IRCCS Materno-Infantile Burlo Garofolo, Trieste, Italy; 70 San Bortolo Hospital, Vicenza, Italy; 71 ARNAS Garibaldi, Catania, Italy; 72 Azienda Ospedaliera di Catanzaro “Pugliese Ciaccio”, Catanzaro, Italy; 73 Niguarda Hospital, Milan, Italy; 74 Ospedale Generale Regionale “F. Miulli” - Acquaviva Delle Fonti, Brindisi, Italy; 75 AO Cà Foncello, Treviso, Italy; 76 Azienda Sanitaria Provinciale di Siracusa, PO Umberto 1°, Syracuse, Italy; 77 Ospedale Fatebenefratelli Isola Tiberina Gemelli Isola, Rome, Italy; 78 Azienda Ospedaliera San Camillo Forlanini, Rome, Italy; 79 Ospedale M. Bufalini, Cesena, Italy; 80 Ospedale “Giovanni Paolo II”, Ragusa, Italy; 81 Central Teaching Hospital of Bolzano, Bolzano, Italy; 82 Azienda Sanitaria Provinciale, Enna, Italy; 83 Istituto Giannina Gaslini, Genoa, Italy; 84 Azienda Ospedaliera Cosenza, Padua, Italy; 85 Fondazione Poliambulanza, Brescia, Italy; 86 Ospedale Buccheri-La Ferla, Palermo, Italy; 87 SS Antonio E Biagio e Cesare Arrigo Hospital, Alessandria, Italy; 88 Panico Hospital, Tricase, LE Italy; 89 S. Spirito Hospital, Pescara, Italy; 90 S. Maria Goretti Hospital, Latina, Italy; 91 Azienda Ospedaliera Universitaria Integrata Di Verona, Verona, Italy; 92 Dipartimento Di Bioetica E Sicurezza, UOS Di Farmacovigilanza, Fondazione Policlinico Universitario A. Gemelli IRCCS - Università Cattolica del Sacro Cuore, Rome, Italy; 93 Department of Woman and Child Health and Public Health – Public Health Area, Fondazione Policlinico Universitario A. Gemelli IRCCS, 00168 Rome, Italy; 94 Sharp Mary Birch Hospital for Women and Newborns, San Diego, CA 92123 USA

**Keywords:** Preterm infants, Lung recruitment, HFOV, INRECSURE, LISA, Surfactant

## Abstract

**Background:**

Surfactant is a well-established therapy for preterm neonates affected by respiratory distress syndrome (RDS). The goals of different methods of surfactant administration are to reduce the duration of mechanical ventilation and the severity of bronchopulmonary dysplasia (BPD); however, the optimal administration method remains unknown. This study compares the effectiveness of the INtubate-RECruit-SURfactant-Extubate (IN-REC-SUR-E) technique with the less-invasive surfactant administration (LISA) technique, in increasing BPD-free survival of preterm infants. This is an international unblinded multicenter randomized controlled study in which preterm infants will be randomized into two groups to receive IN-REC-SUR-E or LISA surfactant administration.

**Methods:**

In this study, 382 infants born at 24^+0^–27^+6^ weeks’ gestation, not intubated in the delivery room and failing nasal continuous positive airway pressure (nCPAP) or nasal intermittent positive pressure ventilation (NIPPV) during the first 24 h of life, will be randomized 1:1 to receive IN-REC-SUR-E or LISA surfactant administration. The primary outcome is a composite outcome of death or BPD at 36 weeks’ postmenstrual age. The secondary outcomes are BPD at 36 weeks’ postmenstrual age; death; pulse oximetry/fraction of inspired oxygen; severe intraventricular hemorrhage; pneumothorax; duration of respiratory support and oxygen therapy; pulmonary hemorrhage; patent ductus arteriosus undergoing treatment; percentage of infants receiving more doses of surfactant; periventricular leukomalacia, severe retinopathy of prematurity, necrotizing enterocolitis, sepsis; total in-hospital stay; systemic postnatal steroids; neurodevelopmental outcomes; and respiratory function testing at 24 months of age. Randomization will be centrally provided using both stratification and permuted blocks with random block sizes and block order. Stratification factors will include center and gestational age (24^+0^ to 25^+6^ weeks or 26^+0^ to 27^+6^ weeks).

Analyses will be conducted in both intention-to-treat and per-protocol populations, utilizing a log-binomial regression model that corrects for stratification factors to estimate the adjusted relative risk (RR).

**Discussion:**

This trial is designed to provide robust data on the best method of surfactant administration in spontaneously breathing preterm infants born at 24^+0^–27^+6^ weeks’ gestation affected by RDS and failing nCPAP or NIPPV during the first 24 h of life, comparing IN-REC-SUR-E to LISA technique, in increasing BPD-free survival at 36 weeks’ postmenstrual age of life.

**Trial registration:**

ClinicalTrials.gov NCT05711966. Registered on February 3, 2023.

**Supplementary Information:**

The online version contains supplementary material available at 10.1186/s13063-024-08240-4.

## Administrative information

Note: The numbers in curly brackets in this protocol refer to the SPIRIT checklist item numbers. The order of the items has been modified to group similar items (see http://www.equator-network.org/reporting-guidelines/spirit-2013-statement-defining-standard-protocol-items-for-clinical-trials/).

**Table:** 

Title {1}	Comparison of “IN-REC-SUR-E” and LISA in preterm neonates with respiratory distress syndrome: a randomized controlled trial (IN-REC-LISA trial)
Trial registration {2a and 2b}	ClinicalTrials.gov Identifier: NCT05711966. Status: recruiting. First Posted: 3/02/2023; Last update posted: 17/02/2023
Protocol version {3}	Version 3, 25 October 2022
Funding {4}	The study has received economic contributions by way of partial coverage of the expenses relating to its implementation
Author details {5a}	Giovanni Vento^1^, Angela Paladini^1^, C. Aurilia^1^, S. Alkan Ozdemir^2^, V.P. Carnielli^3^, F. Cools^4^, S. Costa^1^, F. Cota^1^, C. Dani^5^, P.G. Davis^6^, S. Fattore^1^, C. Fè^1^, N. Finer^7^, F.P. Fusco^1^, C. Gizzi^8^, E. Herting^9^, M. Jian^10^, A. Lio^1^, G. Lista^11^, F. Mosca^12^, S. Nobile^1^, A. Perri^1^, S. Picone^13^, J.J. Pillow^14^, G. Polglase^15^, T. Pasciuto^16^, R. Pastorino^17^, M.Tana^1^, D. Tingay^20^, C. Tirone^1^, A.H. van Kaam^21^, M.L. Ventura^19^, A. Aceti^22^, M. Agosti^23^, G. Alighieri^24^, G. Ancora^25^, V. Angileri^26^, G. Ausanio^27^, S. Aversa^28^, E. Balestri^29^, E. Baraldi^30^, M.C. Barbini^23^, C. Barone^31^, R. Beghini^91^, C. Bellan^32^, A. Berardi^33^, I. Bernardo^27^, P. Betta^35^, M. Binotti^61^, B. Bizzarri^8^, G. Borgarello^36^, S. Borgione^34^, A. Borrelli^27^, R. Bottino^33^, G. Bracaglia^38^, I. Bresesti^23^, I. Burattini^3^, C. Cacace^39^, F. Calzolari^40^, M.F. Campagnoli^41^, L. Capasso^42^, M. Capozza^43^, M.G. Capretti^22^, J. Caravetta^13^, C. Carbonara^41^, V. Cardilli^44^, M. Carta^45^, F. Castoldi^11^, A. Castronovo^8^, E. Cavalleri^28^, F. Cavigioli^11^, S. Cecchi^46^, V. Chierici^23^, C. Cimino^47^, F. Cocca^48^, C. Cocca^48^, P. Cogo^49^, M. Coma^12^, V. Comito^50^, V. Condò^12^, C. Consigli^51^, R. Conti^65^, M. Corradi^40^, G. Corsello^45^, L.T. Corvaglia^22^, A. Costa^52^, A. Coscia^34^, F. Cresi^34^, F. Crispino^27^, P. D’Amico^54^, L. De Cosmo^55^, C. De Maio^36^, G. Del Campo^54^, S. Di Credico^89^, S. Di Fabio^56^, P. Di Nicola^41^, A. Di Paolo^57^, S. Di Valerio^89^, A. Distilo^84^, V. Duca^26^, A. Falcone^50^, R. Falsaperla^47^, V.A. Fasolato^58^, V. Fatuzzo^76^, F. Favini^59^, M.P. Ferrarello^52^, S. Ferrari^59^, F. Fiori Nastro^86^, C.A. Forcellini^91^, A. Fracchiolla^60^, A. Gabriele^72^, F. Galdo^69^, F. Gallini^77^, A. Gangemi^65^, G. Gargano^29^, D. Gazzolo^62^, M.P.Gentile^63^, S.Ghirardello^64^, F. Giardina^65^, L. Giordano^66^, E. Gitto^67^, M. Giuffrè^45^, L. Grappone^48^, F. Grasso^42^, I. Greco^68^, A. Grison^70^, R. Guglielmino^71^, I. Guidotti^33^, I. Guzzo^72^, N. La Forgia^43^, S. La Placa^52^, G. La Torre^74^, P. Lago^75^, L. Lanciotti^3^, A. Lavizzari^12^, F. Leo^29^, V. Leonardi^5^, D. Lestingi^24^, J. Li^10^, P. Liberatore^60^, D. Lodin^76^, R. Lubrano^90^, M. Lucente^84^, S. Luciani^77^, D. Luvarà^90^, G. Maffei^60^, A. Maggio^64^, L. Maggio^78^, K. Maiolo^71^, L. Malaigia^79^, G. Mangili^59^, A. Manna^31^, E.Maranella^56^, A. Marciano^44^, P.Marcozzi^78^, M. Marletta^35^, L. Marseglia^67^, D. Martinelli^74^, S. Martinelli^73^,S. Massari^80^, L. Massenzi^81^, F. Matina^65^, L. Mattia^35^, G. Mescoli^18^, I.V.Migliore^82^, D.Minghetti^83^, I. Mondello^50^, S. Montano^5^, G. Morandi^58^, N. Mores^92^, S. Morreale^82^, I. Morselli^76^, M. Motta^18^, M. Napolitano^31^, D. Nardo^30^, A. Nicolardi^88^, S. Nider^69^, G. Nigro^84^, M. Nuccio^88^, L. Orfeo^77^, C. Ottaviano^78^, P. Paganin^18^, S. Palamides^57^, S. Palatta^57^, P. Paolillo^13^, M.G. Pappalardo^54^, E. Pasta^85^, L. Patti^64^, G. Paviotti^49^, R. Perniola^37^, G. Perotti^64^, S. Perrone^40^, F. Petrillo^53^, M.S. Piazza^82^, A. Piccirillo^66^, M. Pierro^79^, E. Piga^44^, G. A. Pingitore^84^, S. Pisu^16^, C. Pittini^49^, F. Pontiggia^32^, G. Pontrelli^90^, A. Primavera^62^, A. Proto^73^, L. Quartulli^24^, F. Raimondi^42^, L. Ramenghi^83^, M. Rapsomaniki^72^, A. Ricotti^87^, C. Rigotti^19^, M. Rinaldi^60^, F.M. Risso^28^, E. Roma^66^, E. Romanini^40^, V. Romano^77^, E. Rosati^37^, V. Rosella^86^, I. Rulli^67^, V. Salvo^80^, C. Sanfilippo^68^, A. Sannia^36^, A. Saporito^35^, A. Sauna^80^, E. Scapillati^51^, F. Schettini^55^, A. Scorrano^88^, S. Semeria Mantelli^85^, V. Sepporta^26^, P. Sindico^58^, A. Solinas^63^, E. Sorrentino^51^, E. Spaggiari^33^, A. Staffler^81^, M. Stella^79^, D. Termini^86^, G. Terrin^44^, A. Testa^90^, G. Tina^71^, M. Tirantello^76^, B. Tomasini^46^, F. Tormena^75^, L. Travan^69^, D. Trevisanuto^30^, G. Tuling^2^, V. Tulino^39^, L. Valenzano^53^, S. Vedovato^70^, S. Vendramin^75^, P.E. Villani^85^, S. Viola^61^, V. Viola^50^, G. Vitaliti^68^, M. Vitaliti^68^, P. Wanker^81^, Y. Yang^10^, S. Zanetta^61^, E. Zannin^19^ 1. Dipartimento Universitario Scienze della Vita e Sanità Pubblica, UOC di Neonatologia, Fondazione Policlinico Universitario Agostino Gemelli IRCCS—Università Cattolica del Sacro Cuore, Rome, Italy. email: giovanni.vento@unicatt.it; angela.paladini@policlinicogemelli.it; claudia.aurilia@ policlinicogemelli.it; simonetta.costa@ policlinicogemelli.it; francesco.cota@ policlinicogemelli.it; simona.fattore@guest.policlinicogemelli.it; francescapaola.fusco@ policlinicogemelli.it; alessandra.lio@policlinicogemelli.it; stefano.nobile@policlinicogemelli.it; alessandro.perri@policlinicogemelli.it; milena.tana@policlinicogemelli.it; chiara.tirone@policlinicogemelli.it; claudia.fe@ policlinicogemelli.it; 2. Health Science University Dr. Behcet Uz Children’s Hospital, Izmir, Turkey. drsenemalkan@yahoo.com; drtuling@yahoo.com.tr 3. Azienda Ospedaliero Universitaria Ospedali Riuniti, Ancona, Italy. virgilio.carnielli@ospedaliriuniti.marche.it; Ilaria.burattini@ospedaliriuniti.marche.it; Lucia.lanciotti@pm.univpm.it 4. Department of Neonatology, Universitair Ziekenhuis Brussel, Laarbeeklaan 101—1090 Brussel, Belgium. Filip.Cools@uzbrussel.be 5. Department of Mother and Child Health, Division of Neonatology and Neonatal Intensive Care Unit, Careggi University Hospital Florence, Italy. cdani@unifi.it; simona.montano@unifi.it; valentin.leonardi@unifi.it 6. Neonatal Services, Royal Women’s Hospital, Melbourne, Australia. pgd@unimelb.edu.au 7. School of Medicine, University of California, San Diego, San Diego, CA, USA (NNF); and Sharp Mary Birch Hospital for Women and Newborns, San Diego, CA 92123, USA (NNF, AK). nfiner@health.ucsd.edu 8. Unità operativa complessa di neonatologia e TIN Ospedale Sant’Eugenio, ASL ROMA 2, Rome, Italy. camillagizzi@tin.it; bianca.bizzarri@aslroma2.it; antonella.castronovo@aslroma2.it 9. Klinik für Kinder- und Jugendmedizin UNIVERSITÄTSKLINIKUM Schleswig–Holstein Campus Lübeck Ratzeburger Allee 160 │Haus A │23538 Lübeck, Germany. Egbert.Herting@uksh.de 10. Shengjing Hospital of China Medical University, Shenyang, China. lijuan@sj-hospital.org; maojian827@aliyun.com 11. Neonatologia e Terapia Intensiva Neonatale (TIN) Ospedale dei Bambini “V.Buzzi” ASST-FBF-Sacco Via Castelvetro, 32 20,154 Milan, Italy. gianluca.lista@asst-fbf-sacco.it; francesca.castoldi@asst-fbf-sacco.it; francesco.cavigioli@asst-fbf-sacco.it 12. Department of Clinical Sciences and Community Health, University of Milan. Fondazione IRCCS Cà Granda Ospedale Maggiore Policlinico. Via Della Commenda 12 – 20,122 Milan, Italy. fabio.mosca@policlinico.mi.it; Anna.lavizzari@policlinico.mi.it; Martina.coma@unimi.it; Valentina.condo@policlinico.mi.it 13.Policlinico Casilino, Rome, Italy. simpico@libero.it; piermpa@tin.it; jacopo.caravetta@gmail.com 14. Centre for Child Health Research and School of Human Sciences, The University of Western Australia, Perth, WA, Australia. jane.pillow@uwa.edu.au 15. The Ritchie Centre Hudson Institute of Medical Research and Department of Obstetrics and Gynaecology, Monash University, Clayton, 3168 Victoria, Australia. graeme.polglase@monash.edu 16. Research Core Facility Data Collection G-STeP, Fondazione Policlinico Universitario Agostino Gemelli IRCCS, 00168 Rome, Italy. tina.pasciuto@policlinicogemelli.it; simona.pisu@policlnicogemelli.it 17. Section of Hygiene, University Department of Life Sciences and Public Health, Università Cattolica del Sacro Cuore, 00168 Rome, Italy.—roberta.pastorino@policlinicogemelli.it Department of Woman and Child Health and Public Health – Public Health Area, Fondazione Policlinico Universitario A. Gemelli IRCCS, 00168 Rome, Italy 18. Ospedale Maggiore, Bologna, Italy. giovanna.mescoli@ausl.bologna.it; paola.paganin@ausl.bologna.it; mario.motta@ausl.bologna.it 19. Fondazione IRCCS San Gerardo dei Tintori Monza, Italy. mlois.ventura@gmail.com; camilla.rigotti@libero.it; emanuela.zannin@gmail.com 20. Neonatal Research, Murdoch Children’s Research Institute, Melbourne, Australia. David.Tingay@rch.org.au 21. Department of Neonatology, Emma Children’s Hospital, Amsterdam UMC, University of Amsterdam, Vrije Universiteit Amsterdam, Amsterdam, The Netherlands. a.h.vankaam@amsterdamumc.nl 22. Policlinico S. Orsola-Malpighi, Università di Bologna, Bologna, Italy. arianna.aceti2@unibo.it; mariagrazia.capretti@aosp.bo.it; luigi.corvaglia@unibo.it 23. Università degli Studi dell’Insubria, Varese, Italy. massimo.agosti@asst-settelaghi.it; mariacristina.barbini@asst-settelaghi.it; ilia.bresesti@asst-settelaghi.it; valentina.chierici@asst-settelaghi.it 24. P.P. “A. Perrino” Brindisi-ASL BR, Italy. giannialighieri@gmail.com; danila.lestingi@gmail.com; lorenzo.quartulli@asl.brindisi.it 25. Ospedale Infermi, Rimini, Italy. gina.ancora@auslrn.net 26. Presidio Ospedaliero Ingrassia ASP Palermo, Italy. vita.angileri@virgilio.it; vittoriasepporta@yahoo.it; ducavincenzo@alice.it 27. Azienda Ospedaliera S.Anna-S.Sebastiano Caserta, Italy. Gaetano.ausanio@aorncaserta.it; Italo-bernardo@libero.it; ang.bor85@virgilio.it; francescox77@libero.it 28. Azienda Ospedaliera “Spedali Civili”, Brescia, Italy. salvatore.aversa@asst-spedalicivili.it; elicavalleri@gmail.com; francesco.risso@asst-spedalicivili.it 29. Arcispedale Santa Maria Nuova Reggio Emilia, Italy. eleonora.balestri@ausl.re.it; giancarlo.gargano@ausl.re.it; francesco.leo@ausl.re.it 30. University of Padua, Padua, Italy. eugenio.baraldi@unipd.it; daniel.nardo@aopd.veneto.it; Daniele.trevisanuto@gmail.com 31. Ospedale Evangelico Betania-Naples, Italy. cirobarone78@gmail.com; manna.ange@gmail.com; marcello.napolitano@betaniahospital.org 32. Azienda Ospedaliera Bolognini, Seriate (BG), Italy. cristina.bellan@asst.bergamoest.it; federica.pontiggia@gmail.com 33. AOU Policlinico Modena, Italy. alberto.berardi@unimore.it; r.bottino@alice.it; guidotti.isotta@aou.mo.it; spaggiari.eugenio@aou.mo.it 34. Neonatologia Universitaria, Ospedale S.Anna – Città della Salute e della Scienza di Torino, Italy. silvia.borgione@unito.it; Francesco.cresi@unito.it; alessandra.coscia@unito.it 35. AOU Policlinico Vittorio Emanuele-Gaspare Rodolico Catania, Italy. mlbetta@yahoo.it; marisa.marletta@alice.it; lorettamattia@hotmail.com; alessandrosaporito@hotmail.com 36. Azienda Ospedaliera S. Croce e Carle, Cuneo, Italy. borgarello.g@ospedale.cuneo.it; demaio.c@ospedale.cuneo.it; sannia.a@ospedale.cuneo.it 37. Azienda Ospedaliera Vito Fazzi, Lecce, Italy. rperniola@hotmail.com; e.rosati@piafondazionepanico.it 38. Bel Colle Hospital, Viterbo, Italy. giorgio.bracaglia@libero.it 39. Barone Romeo Hospital Patti (ME), Italy. cacacecaterina@gmail.com; vivtul@yahoo.it 40. Azienda OU Parma, Italy. fcalzolari@ao.pr.it. macorradi@ao.pr.it; serafina.perrone@unipr.it; eromanini@ao.pr.it 41. Ospedale PO S. Anna, AOU Città della Salute e della Scienza, Torino, Italy. macampagnoli@cittadellasalute.to.it; pdinicola@cittadelladalute.to.it; ccarbonara@cittadellasalute.to.it 42. Federico II University, Naples, Italy. letizia.capasso@gmail.com; raimondi@unina.it; Fiorentino.grasso89@gmail.com 43. Policlinico di Bari, Italy. manuelacapozza26@gmail.com; Nicola.laforgia@uniba.it 44. Policlinico Umberto I, Rome, Italy. viviana.cardilli@uniroma1.it; Enrico.piga@uniroma1.it; gianluca.terrin@uniroma1.it; alessadra.marciano@uniroma1.it 45. Università degli Studi, Palermo, Italy. mauriziocarta@yahoo.it; giovanni.corsello@unipa.it; mario.giuffre@unipa.it 46. Azienda Ospedaliera Universitaria Senese, Siena, Italy. sara.cecchi@ao-siena.toscana.it; b.tomasini@ao-siena.toscana.it 47. Azienda OU Policlinico “Rodolico-San Marco”, Catania. carla.cimino87@gmail.com; raffaelefalsaperla@hotmail.com 48. Ospedale San Pio, Benevento, Italy. francesco.cocca@ao-rummo.it; carmen.cocca@ao-rummo.it; lidia.grappone@ao-rummo.it 49. Azienda sanitaria universitaria Friuli Centrale, Udine, Italy. paola.cogo@uniud.it; Giulia.paviotti@asufc.sanita.fvg.it; carla.pittini@asufc.sanita.fvg.it 50. AO Bianchi-Melacrino-Morelli Reggio Calabria, Italy. valentina.comito@gmail.com; alessandra.falcone@ospedalerc.it; Isabella.mondello@ospedalerc.it; valeriaviola89@gmail.com 51. S.Pietro Fatebenefratelli, Rome, Italy. chiaraconsigli@yahoo.it; eleonora.scapillati@gmail.com; sorrentino.elena@fbfrm.it 52. Presidio Ospedaliero S. Antonio Abate – Azienda Sanitaria Provinciale, Trapani, Italy. nino8517@hotmail.it; simonalaplaca@gmail.com; Mariapiera84@hotmail.it 53. Di Venere Hospital Bari, Italy. flavia.age@hotmail.it; luigia.valenzano@asl.bari.it 54. Azienda Ospedaliera per l’emergenza Cannizzaro, Catania, Italy; pietrodamico2@gmail.com; giulianadelcampo86@gmail.com; Mgpappalardo02@gmail.com 55. U.O.C T.I.N. e Neonatologia, P.O.C Taranto “Santissima Annunziata”, Italy; lucrezia.decosmo@asl.taranto.it; federico.schettini@uniba.it 56. San Salvatore Hospital L’Aquila, Italy. sandra.difabio57@gmail.com; emaranella@libero.it 57. Azienda Ospedaliera San Giovanni Addolorata, Rome, Italy. adipaolo@hsangiovanni.roma.it; spalamides@hsangiovanni.roma.it; spalatta@hsangiovanni.roma.it 58. Azienda Ospedaliera Carlo Poma Mantova, Italy. valeria.fasolato@asst-mantova.it; grazia.morandi@asst-mantova.it; paola.sindico@asst-mantova.it 59. ASST Papa Giovanni XXIII, Bergamo, Italy. ffavini@asst-pg23.it; sferrari@asst-pg23.it; gmangili@asst-pg23.it 60. Azienda Ospedaliera-Universitaria Ospedali Riuniti Foggia, Italy. fracchiollannalisa@yahoo.it; piolibe@gmail.com; gfmaffei@tiscali.it; matrinaldi@gmail.com 61. Maggiore Hospital Novara, Italy. srnviola@gmail.com; sara.zanetta@gmail.com; marco.binotti@med.uniupo.it 62. Ospedale “Ss Annunziata”, Chieti—Università degli Studi G. D’Annunzio Chieti-Pescara, Italy. dgazzolo@hotmail.com; adeleprimavera@gmail.com 63. Azienza Ospedaliera Universitaria di Ferrara, Italy. m.gentile@ospfe.it; a.solinas@ospfe.it 64. Università di Pavia, Italy. s.ghirardello@smatteo.pv.it; letizia.patti@smatteo.pv.it; al.maggio@smatteo.pv.it; gf.perotti@smatteo.pv.it 65. Ospedale Vincenzo Cervello, Palermo. f.giardina@villasofia.it; f.matina@villasofia.it; a.gangemi@villasofia.it; r.conti@villasofia.it 66. Pineta Grande Castelvolturno (CE), Italy. lucio.giordano@pinetagrande.it; enzaroma@yahoo.it; barry975@libero.it 67. Università degli Studi, Messina, Italy. egitto@unime.it; lmarseglia@unime.it; rulli.imma@tiscali.it 68. ARNAS Civico Hospital Palermo, Italy. Irene.greco@hotmail.it; giulianavitaliti@gmail.com; Sanfilippo.ci@gmail.com; marcello.vitaliti@gmail.com 69. IRCCS materno-infantile Burlo Garofolo, Trieste, Italy. francesca.galdo@burlo.trieste.it; silvia.nider@burlo.trieste.it; laura.tavan@burlo.trieste.it 70. San Bortolo Hospital Vicenza, Italy. alessandra.grison@aulss8.veneto.it; stefania.vedovato@aulss8.veneto.it 71. ARNAS Garibaldi, Catania, Italy. rosannaguglielmino@virgilio.it. kimmaiolo@hotmail.com; gabriella.tina@tiscali.it 72. Azienda Ospedaliera di Catanzaro “Pugliese Ciaccio”, Catanzaro, Italy. immacolata.guzzo@tin.it; maria.rapsomaniki@asp.cz.it; angela.gabriele@asp.cz.it 73. Niguarda Hospital Milan. stefanoenrico.martinelli@ospedaleniguarda.it; alice.proto @ospedaleniguarda.it 74. Ospedale Generale Regionale “F. Miulli”—Acquaviva delle Fonti – Brindisi, Italy. g.latorre@miulli.it; d.martinelli@miulli.it 75. AO Cà Foncello Treviso, Italy. paola.lago@aulss2.veneto.it; francesca.tormena@aulss2.veneto.it; silvia.vendramin@aulss2.veneto.it 76. Azienda Sanitaria Provinciale di Siracusa, PO Umberto 1°, Siracusa, Italy. l_danila@hotmail.com; ezio.morselli@hotmail.it; utin.umbertoprimo@asp.sr.it; valentina.fatuzzo@gmail.com 77. Ospedale Fatebenefratelli Isola Tiberina Gemelli Isola, Rome, Italy. francesca.gallini@fbf-isola.it; stefano.luciani@fbf-isola.it; orfeo@iol.it; valerio.romano@fbf-isola.it 78. Azienda Ospedaliera San Camillo Forlanini, Rome, Italy. lucamaggio@me.com; pmarcozzi1@gmail.com; ottaviano.carla@yahoo.it 79. Ospedale M. Bufalini, Cesena, Italy. laura.malaigia@auslromagna.it; maria.pierro@auslromagna.it; marcello.stella@auslromagna.it 80. Ospedale “Giovanni Paolo II”, Ragusa, Italy. simona.massari@asp.rg.it; vincenzo.salvo@asp.rg.it; alessandra.sauna@asp.rg.it 81. Central Teaching Hospital of Bolzano/Bozen, Italy. lumass2001@yahoo.it; alex.staffler@sabes.it; petra.wanker@sabes.it 82. Azienda Sanitaria Provinciale, Enna, Italy. valentinamigliore@hotmail.it; sabrina.morreale@libero.it; mariasanta.piazza@libero.it 83. Istituto Giannina Gaslini, Genova, Italy. diegominghetti@gaslini.org; luca.ramenghi@unige.it 84. Azienda Ospedaliera Cosenza, Italy. mlucente@aocz.it; gabriellanigro@virgilio.it; antodisti@tiscali.it; Giuli.pingitore@virgilio.it 85. Fondazione Poliambulanza Brescia, Italy. elisa.pasta@poliambulanza.it; simona.semeriamantelli@poliambulanza.it; paolo.villani@poliambulanza.it 86. Ospedale Buccheri-La Ferla, Palermo, Italy. vincenzorosella@libero.it; termini.donatella@fbfpa.it; francescafiorinastro@gmail.com 87. SS Antonio e Biagio e Cesare Arrigo Hospital Alessandria, Italy. aricotti@ospedale.al.it 88. Panico Hospital Tricase (LE), Italy. antonioscorrano@yahoo.it; ales.nicolardi@gmail.com; melissa_nuccio@msn.com 89. S. Spirito Hospital Pescara, Italy. Simona.dicredico@asl.pe.it; Susanna.divalerio@asl.pe.it 90. S. Maria Goretti Hospital Latina, Italy. riccardo.lubrano@uniroma1.it; domenica.luvara@uniroma1.it; giovanna.pontrelli@uniroma1.it; alessiatesta92@live.it 91. Azienda Ospedaliera Universitaria Integrata di Verona, Italy. renzo.beghini@aovr.veneto.it; carloalberto.forcellini@aovr.veneto.it 92. Dipartimento di Bioetica e Sicurezza, UOS di Farmacovigilanza, Fondazione Policlinico Universitario A. Gemelli IRCCS—Università Cattolica del Sacro Cuore, Rome, Italy nadia.mores@policlinicogemelli.it
Name and contact information for the trial sponsor {5b}	The trial does not have a sponsor. The promoter is Fondazione Policlinico Universitario A. Gemelli IRCCS, Università Cattolica del Sacro Cuore, 00168 Rome Italy
Role of sponsor {5c}	The trial does not have a sponsor. There are no obligations towards the promoter other than reporting about the study and study results

## Introduction

### Background and rationale {6a}

Respiratory distress syndrome (RDS) represents the main cause of respiratory insufficiency in preterm infants and is one of the major causes of perinatal morbidity and mortality. Surfactant is a well-established therapy in neonatology. However, the optimal surfactant administration method remains unresolved, especially with the recent clinical focus on avoiding mechanical ventilation in preterm infants born before 28 weeks’ gestational age (i.e., extremely preterm infants). Duration of mechanical ventilation is a key determinant of the severity of bronchopulmonary dysplasia (BPD) [1]. Although attractive and beneficial in clinical practice, the INtubate, SURfactant, Extubate (IN-SUR-E) method cannot be universally applied to all preterm neonates due to non-homogeneous surfactant distribution and lung derecruitment during intubation, resulting in failure of IN-SUR-E in the event of severe RDS. IN-SUR-E also has a failure rate in preterm infants ranging from 19 to 69% [2, 3]. Risk factors for failure of IN-SUR-E are low birth weight, low gestational age, the severity of initial respiratory disease, and a low hemoglobin concentration prior to surfactant administration [2, 4, 5]. A recent randomized clinical trial showed that the application of a recruitment maneuver just before surfactant administration, followed by rapid extubation (INtubate-RECruit-SURfactant-Extubate [IN-REC-SUR-E]), decreased the need for mechanical ventilation during the first 72 h of life compared with IN-SUR-E technique in extremely preterm neonates, without increasing the risk of adverse neonatal outcomes [6]. Recently, a less-invasive surfactant administration (LISA) method was developed with surfactant introduced into the trachea of infants breathing spontaneously using a small catheter instead of an endotracheal tube [7]. The popularity of the LISA technique has increased because it potentially combines the benefits of early surfactant treatment with continuous positive airway pressure (CPAP) and consequent avoidance of mechanical ventilation. The last network meta-analyses on the comparative efficacy of methods for surfactant administration found that among preterm infants, the LISA technique was associated with a lower likelihood of mortality, need for mechanical ventilation, and BPD compared with IN-SUR-E, but these findings did not include a comparison to the IN-REC-SUR-E method [8]. More importantly, data for neonates < 28 weeks’ gestation are not as robust as for the higher gestational age groups due to a smaller number of neonates [9]. Therefore, the safety and efficacy of LISA in this population remain to be confirmed, also considering that extreme prematurity is an independent risk factor for LISA failure [10]. The same authors of meta-analysis agree that data for neonates < 28 weeks are not as robust as for higher gestation age group and that lung recruitment before surfactant administration (IN-REC-SUR-E) represents a promising novel alternative; hence, future randomized evidence directly comparing it to LISA is warranted to draw conclusions concerning the optimal method of surfactant treatment, especially among extremely low gestational age newborns [11]. We therefore designed this study to compare the IN-REC-SUR-E technique with LISA, as recently suggested [11, 12], for evaluating the comparative effectiveness of these techniques in increasing the survival without BPD of extremely preterm infants.

### Objectives {7}

The primary hypothesis of this study is that IN-REC-SUR-E via a high-frequency oscillatory ventilation recruitment maneuver increases survival without BPD at 36 weeks’ postmenstrual age in spontaneously breathing infants born at 24^+0^–27^+6^ weeks’ gestation and failing nasal CPAP or nasal intermittent positive pressure ventilation (NIPPV) during the first 24 h of life compared to LISA treatment.

To confirm this hypothesis, we planned an international multicenter randomized controlled study in which preterm infants will be randomized into two groups: one will receive surfactant with IN-REC-SUR-E modality, and the other one will receive surfactant with LISA treatment.

**Fig. 1:**
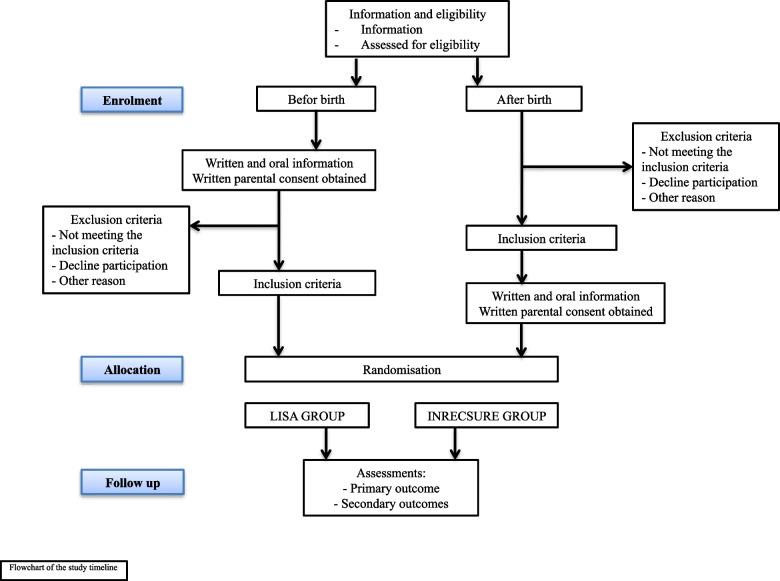
Study flow chart

The study flow chart is detailed in Fig. 1.

### Trial design {8}

The study is an unblinded multicenter randomized superiority trial of the IN-REC-SUR-E vs. LISA technique in infants born at 24^+0^–27^+6^ weeks’ gestation.

The methods of this study are reported according to SPIRIT 2013 Explanation and Elaboration: Guidance for protocols of clinical trials [13].

## Methods: participants, interventions, and outcomes

### Study setting {9}

The following centers are involved in the recruitment for the trial: Fondazione Policlinico Universitario A. Gemelli IRCCS, Università Cattolica del Sacro Cuore, Rome, Italy; S.Pietro Fatebenefratelli, Rome; Fatebenefratelli-Isola Tiberina, Rome; Policlinico Umberto I, Rome; Bel Colle Hospital Viterbo; Fondazione Poliambulanza Brescia; Fondazione MBBM—Ospedale San Gerardo Monza; Niguarda Hospital Milan; Fondazione IRCCS Cà Granda Ospedale Maggiore Policlinico, University Milan; Azienda Ospedaliera Carlo Poma Mantova; SS Antonio e Biagio e Cesare Arrigo Hospital Alessandria; Maggiore Hospital Novara; Azienda Ospedaliera-Universitaria Ospedali Riuniti Foggia; Azienda Ospedaliera Vito Fazzi Lecce; Careggi University Florence; Pineta Grande Castelvolturno; Azienda Ospedaliera S.Anna-S.Sebastiano Caserta; Maggiore Hospital Bologna; AOU Ferrara; AO Cosenza; Di Venere Hospital Bari; Panico Hospital Tricase; Central Teaching Hospital of Bolzano/Bozen; Arcispedale Santa Maria Nuova Reggio Emilia; Barone Romeo Hospital Patti; AOU Policlinico Vittorio Emanuele-Gaspare Rodolico Catania; Università degli Studi di Messina; ARNAS Civico Hospital Palermo; ARNAS Garibaldi, Catania; San Bortolo Hospital Vicenza; AOU Policlinico Modena; San Salvatore Hospital L’Aquila; AO Bianchi-Melacrino-Morelli Reggio Calabria; AO Cà Foncello Treviso; Ospedale PO S. Anna; AOU Città della Salute e della Scienza, Torino; Università di Pavia; Neonatologia Universitaria, Ospedale S.Anna – Città della Salute e della Scienza di Torino; Policlinico Casilino, Rome; Ospedale Evangelico Betania – Napoli; Federico II University, Napoli; University of Padua, Padua; Ospedale San Pio, Benevento; “V.Buzzi” Children’s Hospital, ASST-FBF-Sacco, Milan; Ospedale “Ss Annunziata”, Chieti—Università degli Studi G. D’Annunzio Chieti-Pescara; Azienda Ospedaliera Bolognini, Seriate (BG); ASST Papa Giovanni XXIII, Bergamo; ASST Sette Laghi, Varese, Università degli Studi dell’Insubria; Azienda OU Policlinico “Rodolico-San Marco”, Catania; Policlinico S. Orsola-Malpighi, Università di Bologna, Bologna; Azienda Ospedaliera Universitaria Senese, Siena; Azienda Ospedaliera “Spedali Civili”, Brescia; Azienda Ospedaliera San Giovanni Addolorata, Roma; Azienda Ospedaliera S. Croce e Carle, Cuneo; Azienda Ospedaliera San Camillo Forlanini, Roma; IRCCS materno-infantile Burlo Garofolo, Trieste; Istituto Giannina Gaslini, Genova; Azienda sanitaria universitaria Friuli Centrale, Udine; Università degli Studi, Palermo; Ospedale “Sant’Eugenio”—ASL Roma 2, Roma; Ospedale Infermi, Rimini; Azienda Ospedaliero Universitaria Ospedali Riuniti, Ancona; Ospedale M. Bufalini, Cesena; Ospedale “Giovanni Paolo II”, Ragusa; Ospedale Buccheri-La Ferla, Palermo; Azienda Ospedaliera di Catanzaro “Pugliese Ciaccio”, Catanzaro; Azienda Sanitaria Provinciale di Siracusa, PO Umberto 1°, Siracusa; Presidio Ospedaliero Ingrassia ASP Palermo; Presidio Ospedaliero S. Antonio Abate – Azienda Sanitaria Provinciale, Trapani; Azienda Sanitaria Provinciale, Enna; Ospedale Vincenzo Cervello, Palermo; Azienda Ospedaliera per l’emergenza Cannizzaro, Catania; U.O.C T.I.N. e Neonatologia, P.O.C Taranto “Santissima Annunziata”; Azienda Ospedaliera Universitaria Parma; P.P. “A. Perrino” Brindisi – ASL BR; Università AOUC Policlinico Bari; Ospedale Generale Regionale “F. Miulli”—Acquaviva delle Fonti – BR; Dr. Behcet Uz Children’s Hospital, Izmir, Turkey; The Royal Children’s Hospital, Melbourne, Australia; Shengjing Hospital of China Medical University, Shenyang,China; S. Maria Goretti Hospital Latina; S. Spirito Hospital Pescara; and Azienda Ospedaliera Universitaria Integrata di Verona.

### Eligibility criteria {10}

#### Inclusion criteria

Infants satisfying the following inclusion criteria will be eligible to participate:

1. Born at 24^+0^–27^+6^ in a tertiary neonatal intensive care unit participating in the trial
2. Breathing independently and sufficiently with only nasal CPAP or NIPPV for respiratory support
3. Written parental consent has been obtained
4. Failing nasal CPAP or NIPPV during the first 24 h of life

#### Exclusion criteria

The following are the exclusion criteria:

1. Severe birth asphyxia or a 5-min Apgar score of less than 3
2. Prior endotracheal intubation for resuscitation or insufficient respiratory drive
3. Prolonged (> 21 days) premature rupture of membranes
4. Presence of major congenital abnormalities with possible effects on cardiorespiratory function
5. Hydrops fetalis
6. Inherited disorders of metabolism

### Who will take informed consent? {26a}

The fully trained specific researchers who are on-site will obtain informed prospective consent from participants. Written and oral information will, whenever possible, be offered to parents prior to birth if the mother is at risk for preterm delivery and the infant is likely to be eligible. Informed written consent will be signed by both parents, and sufficient time will be provided for consent. If parents do not speak the local language, consent will only be obtained if an independent interpreter is available. The informed consent will be obtained by the principal investigator of each participating center and their collaborators in charge. A senior investigator will be always available to discuss concerns raised by parents or clinicians during the course of the trial.

### Additional consent provisions for collection and use of participant data and biological specimens {26b}

No additional consents are required. This trial does not involve collecting biological specimens for storage.

### Interventions

#### Explanation for the choice of comparators {6b}

Infants will be allocated to one of the two treatment groups (1:1) according to a restricted randomization procedure [14]. A study investigator (TP) will generate the allocation sequences using both stratification and permuted blocks with random block sizes and block order. The assignment to intervention will be unmasked to all trial participants: parents, research staff, and medical team will be only aware of the study group assignment after randomization procedures.

#### Intervention description {11a}

##### Management in the delivery room

Neonates will be stabilized after birth with positive pressure using a neonatal mask and a T-piece system (for example, Neopuff Infant Resuscitator ®, Fisher and Paykel, Auckland, New Zealand). All neonates will be started on nasal CPAP of at least 6 cmH_2_O via mask or nasal prongs [15]. Newborns who do not breathe or who are persistently bradycardic (heart rate less than 100/min) within the first 60 s after birth will receive positive-pressure ventilation at a rate of 40 to 60 inhalations/min [15] with initial FiO_2_ of 0.30. Infants that will be transitioned successfully to spontaneous breathing will be transferred to the neonatal intensive care unit on nasal CPAP (6–7 cm H_2_O) or NIPPV (PEEP 6–7 cmH_2_O, PIP 12–15 cmH_2_O, respiratory rate 30–40 breaths/min). The decision to intubate and start invasive mechanical ventilation in the delivery room will be in accordance with the American Heart Association Guidelines [16].

The method and timing of umbilical cord clamping will be as per standard practice at each site.

##### CPAP or NIPPV failure criteria

Nasal CPAP or NIPPV will be administered in the neonatal intensive care unit via nasal prongs or nasal mask using the standard method of each participating center, with a pressure of 7–8 cmH_2_O or with a setting of peak inspiratory pressure of 12–15 cmH_2_O, positive end-expiratory pressure of 7–8 cmH_2_O, and rate of 30–40 breaths/min. Infants will receive surfactant with IN-REC-SUR-E or LISA if they need a FiO_2_ of 0.30 or greater to maintain a SpO_2_ between 90 and 94% for at least 30 min, regardless of the non-invasive respiratory support used (CPAP or NIPPV). Infants will also be given surfactant if their clinical status deteriorates rapidly characterized by a rapid increase in oxygen requirements or if they will develop respiratory acidosis defined as a pCO_2_ more than 65 mmHg (8.5 kPa) and a pH less than 7.20, or with lung ultrasound scoring > 8.

A loading dose of intravenous caffeine citrate (20 mg/kg) will be given in the delivery room or immediately after admission to the neonatal intensive care unit (within 2 h of life) and always prior to surfactant administration, followed by a morning intravenous/oral dose of 5–10 mg/kg/day as required.

If a patient, before non-invasive ventilation failure criteria and randomization, will develop severe apnea (more than four episodes of apnea per hour or more than two episodes of apnea per hour requiring ventilation with bag and mask), despite optimal nasal CPAP, nasal intermittent positive pressure ventilation, or bilevel positive airway pressure, the baby will no longer be eligible for the study.

##### Premedications

All neonates of both groups will receive pre-procedural medications, according to local protocols. Suggested schemes are as follows:

- Intravenous atropine (10 µg/kg) in 1 min followed by intravenous fentanyl: 0.5 µg/kg by pump infusion in no less than 5 min (possibly repeatable dose if satisfactory analgosedation is not obtained)

or

- Intravenous atropine (10 µg/kg) in 1 min followed by intravenous ketamine: 0.5 mg/kg in no less than 1 min (possibly repeatable dose if satisfactory analgosedation is not obtained)

The method of premedication will be documented in each participating center.

##### IN-REC-SUR-E group

Infants randomly assigned to the IN-REC-SUR-E group will receive pre-intubation medications and will start after intubation on high-frequency oscillatory ventilation (HFOV) using the following ventilator settings: mean airway pressure 8 cmH_2_O, frequency 15 Hz, and volume guarantee 1.5–1.7 mL/kg [17]. The inspiratory to expiratory ratio will be 1:1. Infants will undergo an oxygenation-guided lung recruitment procedure using stepwise increments and then decrements in the mean airway pressure to recruit and stabilize collapsed alveoli using the de Jaegere method [18]. In particular, optimal recruitment is defined as adequate oxygenation using a FiO_2_ of 0.25 or less. Starting at 8 cmH_2_O, the mean airway pressure will be increased stepwise (2 cmH_2_O every 2–3 min) as long as SpO_2_ improves. The FiO_2_ will be reduced stepwise, keeping SpO_2_ within the target range (90–94%). The recruitment procedure will be stopped if oxygenation no longer improves or if the FiO_2_ is equal to or less than 0.25. The corresponding mean airway pressure will be called the opening pressure. Next, the mean airway pressure will be reduced stepwise (1–2 cmH_2_O every 2–3 min) until the SpO_2_ deteriorates (of at least 2–3 percentage points). The corresponding mean airway pressure will be called the closing pressure. After a second recruitment maneuver at the opening pressure for 2 min, the optimal mean airway pressure will be set at 2 cmH_2_O above the closing pressure for at least 3 min. Immediately after the recruitment procedure, infants in the IN-REC-SUR-E group will receive 200 mg/kg of poractant alfa (Chiesi Farmaceutici S.p.A., Parma, Italy) via a closed administration system in one or two aliquots, while continuing high-frequency oscillatory ventilation. Infants with sufficient respiratory drive and stable clinical conditions will be extubated within 30 min after surfactant administration irrespective of the FiO_2_ and will recommence on nasal CPAP (7–9 cm H_2_O) [19] or NIPPV. Cases of failure to extubate due to complications or intercurrent conditions will be reported, defined, and included in the statistical analysis.

##### LISA group

By contrast, infants allocated to the LISA group will receive 200 mg/kg of poractant alfa (Chiesi Farmaceutici S.p.A., Parma, Italy) according to the following protocol: during nasal CPAP with a pressure of 7–8 cm H_2_O, surfactant will be administered over 0.5–3 min using the SurfCath™ tracheal instillation catheter (VYGON S.A. – Ecouen, France), or a 4–6-F end-hole catheter, according to local protocols. After pre-procedural medications, the catheters will be positioned during laryngoscopy with or without Magill forceps. The catheter will be connected to a syringe pre-filled with the surfactant, and the surfactant is instilled slowly. The infant’s mouth will be closed. In cases of apnea or bradycardia, positive pressure ventilation will be performed until recovery by nasal prongs or mask or by endotracheal tube if necessary. After surfactant administration, CPAP (7–9 cm H_2_O) [19] or NIPPV will be provided unless failure criteria are met (see below).

Transcutaneous PaCO_2_ will be recorded during surfactant administration in both procedures (IN-REC-SUR-E and LISA), if available. Changes in respiratory support settings are permitted to maintain transcutaneous CO_2_ values in each site’s accepted range.

In both groups, maintaining a FiO_2_ < 0.30 to obtain SpO_2_ values in the desired range (90–94%) will drive weaning of the level of CPAP or in the rate of NIPPV in the following days. In the babies managed with CPAP, the decision as to whether to begin bilevel-positive airway pressure or NIPPV to prevent the need for re-intubation in infants of both groups will be up to the neonatologist on duty and will be considered in the final analysis.

Infants in both groups who meet the CPAP/NIPPV failure criteria again during the following 24 h will receive a second dose of surfactant (100 mg/kg of poractant alfa) according to the randomized group (IN-REC-SUR-E or LISA). The minimum time interval between the first and second doses of surfactant is 6 h. In case of respiratory deterioration requiring endotracheal intubation immediately after the first surfactant administration, this will be interpreted as extubation failure in the INRECSURE group or as severe respiratory failure requiring intubation in the LISA group.

The indications for invasive mechanical ventilation via endotracheal tube after IN-REC-SUR-E or LISA will be as follows:

- Poor oxygenation with FiO_2_ above 0.40 for more than 6 h to maintain a SpO_2_ between 90 and 94% despite CPAP (7–9 cm H_2_O) or NIPPV (with peak inspiratory pressure of 15–20 cmH_2_O, positive end-expiratory pressure of 6–8 cmH_2_O, and rate of 40–60 breaths/min)
- Respiratory acidosis (either capillary pCO_2_ or PaCO_2_ > 65 mm Hg [8.5 kPa] and pH < 7.20)
- Apnea (more than four episodes of apnea per hour or more than two episodes of apnea per hour requiring ventilation with bag and mask), despite optimal nasal CPAP, nasal intermittent positive pressure ventilation, or bilevel positive airway pressure

#### Criteria for discontinuing or modifying allocated interventions {11b}

On the consent form, participants are informed that they can withdraw from the study at any time without losing any rights to treatment, either current or future. Clinical data will be destroyed only if the right to be forgotten will be requested according to GDPR [ref: https://gdpr-info.eu/art-17-gdpr/]. In this case, the record ID and the allocation arm will be traced, and the reason for withdrawal will be recorded in the eCRF. The trial may be stopped if during the trial or after interim analysis, unwanted effects have occurred, new information becomes available and the experimentation is no longer in the best interests of this population, the agreed rules for participation in the trial are not followed, and the trial is interrupted by the component authorities or by the promoter.

#### Strategies to improve adherence to interventions {11c}

Given the nature of the intervention only after adherence to the protocol by the participating centers, no specific strategy is envisaged to improve intervention adherence.

#### Relevant concomitant care permitted or prohibited during the trial {11d}

For ethical reasons, all types of care as usual are permitted during the trial.

#### Provisions for post-trial care {30}

There will not be a specific post-trial care. No harm from participation in the trial is expected. All preterm infants enrolled in the trial, after discharge, will start a “preterm-follow-up program” as a normal clinical practice for all preterm discharged from hospitals participating in the study. Infants enrolled in trials will be tested for neurodevelopmental outcomes and respiratory function at 24 months, the last of the secondary outcomes (see section below).

### Outcomes {12}

#### Primary outcome measure

A composite outcome of death or bronchopulmonary dysplasia (BPD) at 36 weeks’ postmenstrual age [20] is the primary outcome because BPD represents the most severe respiratory morbidity of preterm infants, and death is a competing risk. The diagnosis of BPD will be ascertained by a standardized test [21]. Infants remaining on mechanical ventilation or CPAP at 36 weeks’ postmenstrual age, or those with a supplemental oxygen concentration ≥ 0.30 to obtain SpO_2_ between 90 and 94% will receive a BPD diagnosis without additional testing. Infants with a supplemental oxygen concentration < 0.30 to obtain SpO_2_ between 90 and 94% or those receiving high-flow nasal cannula therapy will undergo a timed stepwise reduction to room air without any flow. Those in whom the reduction will not be tolerated will receive a BPD diagnosis.

#### Secondary outcome measures

The following are the secondary outcome measures:

1. BPD at 36 weeks’ postmenstrual age.
2. Grade of BPD at 36 weeks’ postmenstrual age.
3. Death at 36 weeks’ postmenstrual age or before discharge.
4. SpO_2_/FiO_2_ at 3 days, 7 days, and thereafter every 7 days until 36 weeks’ postmenstrual age [22]
5. Severe intraventricular hemorrhage (IVH) (grade 3 or 4 based on the Papile criteria) [23].
6. Occurrence of air leaks including pneumothorax or pulmonary interstitial emphysema before discharge.
7. Need and duration of invasive respiratory support.
8. Duration of non-invasive respiratory support.
9. Duration of oxygen therapy.
10.  Pulmonary hemorrhage.
11.  PDAhs (patent ductus arteriosus; hemodynamically significant), i.e., requiring pharmacological treatment with ibuprofen/indomethacin/acetaminophen).
12.  Percentage of infants receiving two or more doses of surfactant.
13.  Incidence of PVL (periventricular leukomalacia) [24].
14.  Incidence of ROP (retinopathy of prematurity) grade 3 or above [25].
15.  Incidence of NEC (necrotizing enterocolitis) grade 2 or above [26].
16.  Incidence of sepsis defined as a positive blood culture or suggestive clinical and laboratory findings leading to treatment with antibiotics for at least 7 days despite the absence of a positive blood culture.
17.  Total in-hospital stay.
18.  Use of systemic postnatal steroids.
19.  Neurodevelopmental outcomes via Bayley scales of infant development-III and respiratory function testing at 24 months of age. In particular, lung function tests will be performed at 2 years of life according to current American Thoracic Society (ATS)/European Respiratory Society (ERS) guidelines. Tidal breathing flow volume loop (TBFVL) and multiple-breath nitrogen washout (MBNW) will be performed during spontaneous sleep. An appropriately sized face mask will be gently placed covering the mouth and nose of infants lying supine. The reported MBNW outcomes will be the functional residual capacity (FRC) and the lung clearance index (LCI), which represents a measure of the number of times the volume of gas in the lung at the start of the washout (the FRC) must be turned over in order to wash out the tracer to the pre-defined endpoint. Pulmonary function testing will be offered to all infants in selected centers distributed in different geographic areas, equipped with specific devices and with expertise in data interpretation.

### Other collected data

The following data will be recorded for each infant: gestational age (GA), birth weight (BW), BW *z* score, sex, Apgar score at 5 min, antenatal steroid treatment (complete course), preterm PROM > 18 h, diagnosis of clinical chorioamnionitis (defined as maternal fever, uterine tenderness, abdominal pain, foul-smelling vaginal discharge, maternal and fetal tachycardia, elevated white blood cell count), maternal hypertension disorders, and type of delivery.

### Participant timeline {13}

All participants will complete the same outcome assessments as presented in Fig. 1 and Table 1.

**Table 1:** Overview of enrolment, interventions, and assessments

	Study period					
	Enrollment	Allocation	Post-allocation			Close-out
Time point	* − t*_*1*_	0	*t*_*1*_	*t*_2_*	*t*_*3*_	*t*_*4*_
Enrollment						
Eligibility screen	X					
Informed consent	X					
Allocation		X				
Interventions						
*INRECSURE group*			X	X	X	X
*LISA group*			X	X	X	X
Assessments						
*Information on background and randomization*	X					
*Primary outcome*					X	
*Secondary outcomes*			X	X	X	X
*Other collected data*	X					

− *t*_*1*_ before or after birth, *0* at the time of allocation, *t*_*1*_ first surfactant administration within 2 h of life, *t*_2_* possible second dose of surfactant during the following 24 h after the first dose, *t*_*3*_ at discharge or death, *t*_*4*_ follow up at 24 months of age

### Sample size {14}

In order to assess the superiority of the IN-REC-SUR-E with respect to the LISA technique, we hypothesized that employing the IN-REC-SUR-E technique for surfactant administration in extremely preterm infants could lead to an increased survival rate without BPD at 36 weeks of postmenstrual age compared to the LISA approach, raising it from 65 to 80%. Our estimation of a 15% difference is grounded in data from the German Neonatal Network (GNN) regarding the LISA approach, findings from the recently published OPTIMIST trial [27], and updated information on the IN-REC-SUR-E technique from select Italian centers, which continued its use post the conclusion of the INRECSURE study. To achieve 90% statistical power at a 0.05 significance level, we determined that 181 newborns must be enrolled in each group. Factoring in a 5% risk of including patients who do not meet the inclusion criteria after randomization, a total of 382 patients will be randomly assigned. Twins will be randomized separately.

### Recruitment {15}

The obstetricians are aware of the study protocol and will inform the neonatologists of any case of high-risk preterm birth. Written and oral information will, whenever possible, be offered to parents prior to birth if the mother is at risk for preterm delivery and the infant is likely to be eligible. In the few cases of spontaneous preterm labor and consequent vaginal delivery, the informed consent will be obtained soon after the birth.

A monthly accrual report about the study will be sent to participating centers.

### Assignment of interventions: allocation

#### Sequence generation {16a}

Infants will be allocated to one of the two treatment groups (1:1) according to a restricted randomization procedure [14]. A biostatistician (TP) will generate the allocation sequences using both stratification and permuted blocks with random block sizes and block order. Stratification factors will include center and gestational age (24^+0^ to 25^+6^ weeks or 26^+0^ to 27^+6^ weeks).

#### Concealment mechanism {16b}

The table of allocation will not be disclosed to ensure concealment, and the randomization will be provided through the Research Electronic Data Capture (REDCap) web application (https://redcap-irccs.policlinicogemelli.it/). The assignment to intervention will be unmasked to all trial participants: parents, research staff, and medical team will be only aware of the study group assignment after randomization procedures.

#### Implementation {16c}

Enrollment and assigning participants to interventions will be performed as previously described.

### Assignment of interventions: blinding

#### Who will be blinded {17a}

Except for the biostatistician involved in the analysis, the assignment to intervention will be unmasked to all trial participants: parents, local site research staff, and medical team will be only aware of the study group assignment after randomization procedures.

#### Procedure for unblinding if needed {17b}

Refer to above {17a}.

##### Data collection and management

#### Plans for assessment and collection of outcomes {18a}

Local principal investigators are required to participate in preparatory meetings in which details of the study protocol, data collection, and IN-REC-SUR-E and LISA procedures will be accurately discussed. All centers will receive detailed written instructions on web-based data recording, and, to resolve any difficulties, it will be possible to contact the Research Core Facility Data Collection (Fondazione Policlinico Universitario A. Gemelli IRCCS, Università Cattolica del Sacro Cuore). Moreover, it will be ascertained that IN-REC-SUR-E and LISA procedures are followed similarly in all participating centers.

#### Plans to promote participant retention and complete follow-up {18b}

Each participating investigator will be responsible for ensuring data quality. Each reported information will be systematically checked for consistency, completeness, data processing, and monitoring. All study data will be (1) screened for out-of-range data, with cross-checks for conflicting data within and between data collection forms by a data manager, and (2) referred back to the relevant center for clarification in the event of missing items or uncertainty.

The data manager will keep a record of all discrepancies and resolutions. The chief investigator and trial statistician will review the results generated for logic and for patterns or problems. Outlier data will be investigated.

A monthly accrual report about the study will be sent to participating centers.

#### Data management {19}

A customized electronic case report form (eCRF) will be created for the study. Data processing will take place in compliance with current Italian and European legislation regarding the General Data Protection Regulation. Data sharing with non-European Union centers will be performed according to Standard Contract Clauses. Each participating center will be identified by a three-digit code, and within each center, patients will be identified with a progressive number. Pseudo-anonymized study data will be collected and managed using REDCap electronic data capture tools hosted at Fondazione Policlinico Universitario A. Gemelli, IRCCS (https://redcap-irccs.policlinicogemelli.it/). REDCap is a secure, web-based application designed to support data capture for research studies, providing (1) an intuitive interface for validated data entry, (2) audit trails for tracking data manipulation and export procedures, (3) automated export procedures for seamless data downloads to common statistical packages, and (4) procedures for importing data from external sources [28, 29]. To prevent possible data entry mistakes and to improve data quality, the eCRF will be implemented by design according to validation, branching, and skipping logic quality criteria.

#### Confidentiality {27}

Only people officially registered as study investigators or data managers will receive a user login to access the REDCap web platform and enter/manage data. All collected data will be obtained from the clinical records. Each participating center must maintain appropriate medical and research records for this trial and regulatory/institutional requirements for the protection of the confidentiality of study subjects. The principal investigator is responsible for assuring that the data collected are complete, accurate, and recorded in a timely manner. Clinical information will be collected at the following times:

1. At trial entry: information on eligibility, background information, and randomization
2. Following randomization: all data above listed in the “Primary outcome measure”, “Secondary outcome measure”, and “Other collected data” sections.

Further information will be collected on expected serious adverse events.

#### Plans for collection, laboratory evaluation, and storage of biological specimens for genetic or molecular analysis in this trial/future use {33}

This trial does not involve collecting biological specimens for storage.

## Statistical methods

### Statistical methods for primary and secondary outcomes {20a}

Analyses will adhere to both the intention-to-treat and per-protocol principles, following the recommendations outlined in the CONSORT guidelines, with the primary outcome evaluated in the intention-to-treat population [30]. The intention-to-treat group will encompass all participants assigned to the study intervention, while the per-protocol population will comprise individuals who receive and complete the study intervention, meeting all the study criteria.

For the primary outcome, a log-binomial regression model will be employed, adjusting for the stratification factors of gestational age and study center to estimate the adjusted relative risk (RR). Additionally, we will calculate the absolute risk reduction and the number-needed-to-treat. Statistical analyses will be conducted using the Stata software, version 16.

### Interim analyses {21b}

An interim analysis for safety to evaluate the prespecified stopping rules will be done at 30% and 60% of recruitment by an independent statistician, masked to the treatment allocation. The prespecified clinical and safety stopping rules will be in-hospital mortality rate of more than 40%, a rate of severe intraventricular hemorrhage of more than 30%, and a pneumothorax rate of more than 10%, considering overall occurrences. The data and safety monitoring board will have unmasked access to all data and will discuss the interim analysis results with the steering committee in a joint meeting. The steering committee will then determine the trial’s continuation and report to the central ethics committee.

### Methods for additional analyses (e.g., subgroup analyses) {20b}

Subgroup analyses will be conducted to explore specific factors such as gestational age influencing the outcomes, providing a more nuanced understanding of the intervention’s effects within distinct subpopulations.

### Methods in analysis to handle protocol non-adherence and any statistical methods to handle missing data {20c}

Considering the variables collected and the planned control in the data collection procedure, we do not anticipate a high percentage of missing data. Each reported information will be systematically checked for consistency, completeness, data processing, and monitoring (refer to paragraph **{**18b**}**). For these reasons, no imputation will be provided for missing data.

### Plans to give access to the full protocol, participant-level data, and statistical code {31c}

The datasets analyzed during this trial are available from the corresponding author upon reasonable request.

### Duration of study

In this study, 382 infants will be recruited. The trial will terminate when the last recruited infant discharged from the hospital will be evaluated for neurodevelopmental outcome and respiratory function at 24 months of follow-up. The planned duration for randomization is 3 years.

### Oversight and monitoring

#### Composition of the coordinating center and trial steering committee {5d}

A joint Trial Steering Committee (TSC) and Data and Safety Monitoring Board (DSMB) will provide supervision for the trial, providing advice to the chief and co-investigators on all aspects of the trial conduct and affording protection for patients by ensuring the trial is conducted according to the Guidelines for Good Clinical Practice in Clinical Trials. The chief investigator and advisory board will chair the TSC. A trial manager, a statistician, and other investigators will form the DSMB. The trial manager and other investigators will check randomly the accuracy and consistency of the data entered from the participating centers. The data will be reported to the PI and advisory board as “group A” and “group B.” An independent statistician, blinded to the treatment allocation, will perform the interim analysis. The statistician will report to the DSMB. The DSMB will have unblinded access to all data and will discuss the results of the interim analysis with the Steering Committee in a joint meeting. The Steering Committee will decide on the continuation of the trial and will report to the central Ethics Committee.

#### Composition of the data monitoring committee, its role, and reporting structure {21a}

Refer to above {5d}.

#### Adverse event reporting and harms {22}

Adverse events, device deficiency, and incidents, as identified by the following definitions, will be recorded and reported to the Manufacturer and the National Competent Authority as per applicable law (for EU Centers Regulation 2017/745 and Centers in Italy Circular of the Ministry of Health Application of the EU Regulation 2017/745 of the European Parliament and of the Council, of 5 April 2017, in the field of clinical investigations relating to medical devices).

Site investigators will report any adverse event to the coordinating center. In case of a serious event involving a medical device, serious incident, a report (for centers located in Italy from Dispovigilance—https://www.salute.gov.it/DispoVigilancePortaleRapportoOperatoreWeb/) will be issued and sent to the Competent Authority, the manufacturer, and the coordinating center, as per national applicable law. Device deficiency will be reported by the investigators to the coordinating center and to the manufacturer, as per applicable law.

Adverse events, device deficiency, and incidents will be identified by the following definitions:

- *Incident* means any malfunction or deterioration in the characteristics or performance of a device made available on the market, including use error due to ergonomic features, as well as any inadequacy in the information supplied by the manufacturer and any undesirable side effect; “serious incident” means any incident that directly or indirectly led, might have led, or might lead to any of the following: (a) the death of a patient, user, or other persons; (b) the temporary or permanent serious deterioration of a patient’s, user’s, or other persons’ state of health; and (c) a serious public health threat.
- *Device deficiency* means any inadequacy in the identity, quality, durability, reliability, safety, or performance of an investigational device, including malfunction, use errors, or inadequacy in information supplied by the manufacturer.
- *Adverse event* means any untoward medical occurrence, unintended disease or injury, or any untoward clinical signs, including an abnormal laboratory finding, in subjects, users, or other persons, in the context of a clinical investigation, whether or not related to the investigational device.
- *Serious adverse event (SAE)* means any adverse event temporally related to the procedure that led to any of the following situations during hospitalization: (a) death and (b) serious deterioration in the health of the subject that resulted in any of the following: (i) life-threatening illness or injury, (ii) permanent impairment of a body structure or a body function, (iii) hospitalization or prolongation of patient hospitalization, (iv) medical or surgical intervention to prevent life-threatening illness or injury or permanent impairment to a body structure or a body function, and (v) chronic disease secondary to adverse events related to the procedure.

Safety end-point measures will include incidence, severity, and causality of reported serious adverse effects, namely changes in the occurrence of the expected common complications of prematurity and clinical laboratory test assessments and the development of unexpected SAEs in this high-risk population. All SAEs will be graded according to Common Terminology Criteria for Adverse Events (CTCAE) version 5.0. All SAEs and serious incidence (SI) will be followed until satisfactory resolution or until the investigator responsible for the care of the participant deems the event to be chronic or the patient to be stable. Particular attention will be paid to the SAEs (in-hospital mortality, serious intraventricular hemorrhage, and pneumothorax) considered stopping rules. They have to be inserted by local investigators in the eCRF and reported to the PI and advisory board as soon as possible, no later than 24 h after their occurrence.

#### Frequency and plans for auditing trial conduct {23}

A monthly accrual report about the study will be sent to participating centers.

Whenever a SAE/SI will be registered in REDcap’s eCRF, a progressive ID will be attributed, and a report will be sent to the local principal investigator in order to verify the appropriateness of the data and to facilitate the notification to the chief investigators according to a standardized template. More in-depth, all expected and unexpected SAEs/SIs, whether or not they are attributable to the study intervention, will be reviewed by the local principal investigators to determine if there is reasonable suspected causal relationship to the intervention. Revised data will be reported to chief investigators (in-rec-lisa@policlinicogemelli.it) who will report to the Ethics Committee and inform all investigators to guarantee the safety of participants. The DSMB will be responsible for monitoring the adverse events.

#### Plans for communicating important protocol amendments to relevant parties (e.g., trial participants, ethical committees) {25}

Important protocol modifications will be reported to and need to be approved by all the medical-ethical committees. When modifications are approved, these will also be reported to the participants and be added to the *Trials* paper and the study registration on ClinicalTrials.gov.

#### Dissemination plans {31a}

The outcomes of this study about the effectiveness of the intervention will be reported in article(s) in international peer-reviewed journals. Both positive and negative outcomes will be reported. The outcomes will also be reported in professional magazines in the field and in magazines for parents. The results will be presented in participating care organizations and at scientific conferences. All presentations and publications are expected to protect the integrity of the major objectives of the study; data that break the blind will not be presented prior to the release of mainline results.

Recommendations as to the timing of the presentation of such endpoint data and the meetings at which they might be presented will be given by the Steering Committee. Substantive contributions to the design, conduct, interpretation, and reporting of the clinical trial will be recognized through the granting of authorship on the final trial report. Individuals who fulfill the authorship criteria will have final authority over the manuscript content.

## Discussion

The primary hypothesis of this trial is that surfactant administration by IN-REC-SUR-E, via a high-frequency oscillatory ventilation recruitment maneuver, increases survival without BPD at 36 weeks of postmenstrual age in spontaneously breathing infants born at 24^+0^–27^+6^ weeks’ gestation affected by RDS, compared to LISA.

Although surfactant replacement therapy is an established treatment in infants with RDS, identifying the safest and most effective way to administer surfactant is critically important but not well defined, especially in extremely preterm infants. It is plausible to expect that the therapeutic benefits of exogenous surfactant therapy will be maximized by quickly and uniformly aerating the lung beforehand. The IN-SUR-E method cannot be universally applied to all preterm neonates; risk factors for failure of IN-SUR-E are low birth weight, low gestational age, the severity of initial respiratory disease, and a low hemoglobin concentration prior to surfactant administration [2, 4, 5]. New promising evidence from the randomized clinical trial INRECSURE showed that the application of a recruitment maneuver just before surfactant administration, followed by rapid extubation, decreased the need for mechanical ventilation during the first 72 h of life compared with the IN-SUR-E technique in extremely preterm neonates, without increasing the risk of adverse neonatal outcomes [6]. The recent LISA technique introduces surfactant into the trachea of spontaneously breathing infants via a small catheter instead of an endotracheal tube [7]. LISA is now widespread and combines the benefits of early surfactant treatment with CPAP and consequent avoidance of mechanical ventilation. The last network meta-analyses on the comparative efficacy of methods for surfactant administration found that among preterm infants, the LISA technique was associated with a lower likelihood of mortality, need for mechanical ventilation, and BPD compared with IN-SUR-E, but these findings did not include the comparison to IN-REC-SUR-E method [8]. More importantly, data for neonates < 28 weeks’ gestation are not as robust as for the higher gestation age groups due to a smaller number of neonates [9]. For these reasons, the safety and efficacy of LISA in infants < 28 weeks remain to be confirmed, and lung recruitment before surfactant administration (IN-REC-SUR-E) represents a promising new alternative. We therefore designed this study to compare the IN-REC-SUR-E technique with LISA, for evaluating the comparative effectiveness of these techniques in increasing the survival without BPD of extremely preterm infants.

## Trial status

The trial is currently recruiting study subjects.

### Supplementary Information

Supplementary Material 1.

Supplementary Material 2.

## Data Availability

The datasets generated and/or analyzed during the study are available from the corresponding author upon reasonable request.

## Abbreviations

BPD: Bronchopulmonary dysplasia
BW: Birth weight
CTCAE: Common Terminology Criteria for Adverse Events
CPAP: Continuous positive airway pressure
DSMB: Data and Safety Monitoring Board
eCRF: Electronic case report form
FiO_2_: Fraction of inspired oxygen
GA: Gestational age
GNN: German Neonatal Network
HFOV: High-frequency oscillatory ventilation
IN-REC-SUR-E: INtubate-RECruit-SURfactant-Extubate
IN-SUR-E: Intubate-Surfactant-Extubate
IVH: Intraventricular hemorrhage
LISA: Less-invasive surfactant administration
nCPAP: Nasal continuous positive airway pressure
NEC: Necrotizing enterocolitis
NICU: Neonatal intensive care unit
NIPPV: Nasal intermittent positive pressure ventilation
PDA: Patent ductus arteriosus
PDAhs: Hemodynamically significant patent ductus arteriosus
PROM: Premature rupture of membranes
PVL: Periventricular leukomalacia
RedCap: Research Electronic Data Capture
RDS: Respiratory distress syndrome
ROP: Retinopathy of prematurity
RR: Relative risk
SpO_2_: Pulse oximetry
SAE: Serious adverse event
SI: Serious incidence
TSC: Trial Steering Committee
