# Supplementary material for: Comparison of “IN-REC-SUR-E” and LISA in preterm neonates with respiratory distress syndrome: a randomized controlled trial (IN-REC-LISA trial)
Source: Trials. 2024 Jul 2;25:433. doi: 10.1186/s13063-024-08240-4 (PMC11218154; doi:10.1186/s13063-024-08240-4)
Supplement: Supplementary file 1 — Supplementary Material 1. [file 13063_2024_8240_MOESM1_ESM.docx]

**INFORMATION SHEET FOR PARTICIPATION IN A CLINICAL TRIAL AND DECLARATION OF CONSENT FOR**

**PARENTS / GUARDIANS**

**TITLE: “COMPARISON BETWEEN“ IN-REC-SUR-LE ”> - AND“ LISA ”IN PREMATURE INFANTS WITH RESPIRATORY DISTRESS SYNDROME: A CONTROLLED RANDOMIZED STUDY (IN-REC-LISA)”.**

OPERATIVE UNIT ….
EXPERIMENTER: Prof. …….
TELEPHONE: ……..

Dear Mrs. / Mr.

the information contained in the following information sheet is very detailed. We ask you to make the decision to accept or refuse to let your child / protege participate ONLY after reading this sheet and having a FULL INTERVIEW with a member of the testing group who will have to take the NECESSARY TIME to fully understand what is proposed to her.

It is your right to be informed about the purpose and characteristics of the trial so that you can make an informed and free decision whether to involve your child / protégé.

This document aims to inform you about the nature of the trial, about the purpose it proposes, about what participation will entail for your child / protégé, including your rights and responsibilities.

Please read the following carefully. The researchers involved in this project, indicated at the beginning of this document, are available to answer your questions. No question that comes to your mind is trivial: don't be afraid to ask it!

In addition to us, you can discuss the proposal contained in this document with your family doctor, your family and other people you trust. Take your time to decide. You can take home an unsigned copy of this document to think about it or to discuss it with others before making a decision. If you decide not to have your child / protégé participate in the trial, you will still receive the best possible care for patients with your child's / protégé's condition / illness.

Your refusal will in no way be interpreted as a lack of trust.

The Principal Investigator

**INFORMATION SHEET**

Dear Parent / Guardian,

A study entitled "Comparison between" IN-REC-SUR-E "> - and" LISA "in premature infants with respiratory distress syndrome: a randomized controlled study (IN -REC-LISA) ".

This research is international in nature - multicentre and aims to compare the effectiveness of two surfactant administration techniques for the treatment of patients suffering from the same disease from which your child / child is affected.

To carry out this research we would like to make use of the collaboration and availability of people who, like your child / protege, meet the scientific requirements suitable for the assessment that will be carried out. Whether or not you decide to participate in this study will have no impact on the care your child / protector will receive and doctors will continue to follow you with due care.

However, before you make the decision to accept or refuse to participate, please read these pages carefully, taking all the time you need, and ask for clarification if you do not understand or need further clarification. Furthermore, if you wish, you can ask your family members or a trusted doctor for an opinion before deciding.

**WHAT THE STUDY IS ABOUT**

The study aims to compare the efficacy of two surfactant administration techniques: the IN-REC-SUR-E (INtubate-RECruit-SURfactant-Extubate) technique and the LISA (Less Invasive Surfactant Administration) technique.

Surfactant is a well-established therapy in neonatology for the treatment of respiratory distress syndrome (RDS), but the optimal method of administration is still under study. A recent randomized clinical trial showed that the application of a Lung recruitment just prior to surfactant administration, followed by rapid extubation (IN-REC-SUR-E) was shown to be safe and reduced the need for mechanical ventilation during the first 72 hours of life in extremely premature infants compared to the IN technique -SUR-E (INtubation-SURfactant-Extubation) which involves the administration of the surfactant by transient intubation without recruitment maneuver.

In recent years, a surfactant administration method (LISA) has been developed which does not involve intubation but the use of a small dedicated catheter instead of the endotracheal tube for the administration of surfactant into the trachea of ​​spontaneously breathing newborns. The potential advantages of the LISA technique are related to the benefits of surfactant treatment with continuous positive airway pressure (CPAP) via the nose, avoiding the use of invasive mechanical ventilation.

To date, there are no studies comparing the IN-REC-SUR-E technique and the LISA technique in extremely premature infants (i.e. with a gestational age of less than 28 weeks). We therefore designed this study to compare the two techniques in order to evaluate their effectiveness in increasing survival without developing pulmonary bronchodysplasia (BPD), which is the most frequent complication of neonatal respiratory failure.

The hypothesis of this study is that the IN-REC-SUR-E technique, by means of a pulmonary recruitment maneuver performed with high-frequency oscillatory ventilation, increases the survival without BPD at the post-conception age of 36 weeks in preterm infants born. at 24 + 0 27 + 6 weeks of gestational age, affected by RDS, who breathe spontaneously and who fail nasal CPAP treatment, requiring surfactant therapy during the first 24 hours of life, compared to treatment with LISA technique.

**WHAT DOES THE PARTICIPATION OF YOUR SON / PROTECTED IN THE STUDY INVOLVE**

In case you decide to involve your child / protege in the study, the experimental design of this research foresees that one group of patients will receive the administration of surfactant using the IN-REC-SUR-E technique and a second group will receive the administration of surfactant. using the LISA technique.

The study is randomized, i.e. the assignment to one of the above treatment groups will follow a statistical criterion.

The study will last 3 years and 381 patients will participate in this research, chosen among all those who were born at 24 + 0 - 27 + 6 weeks of gestation and affected by the same disease as your child / guardian.

If you agree to have your child / protege participate in this study, he will undergo a first visit to verify that his conditions meet the criteria required by the study.

Participation in the trial does not entail any increase in expenses for your child / protected person, nor will it provide for any compensation.

**WHAT ARE THE RISKS ARISING FROM PARTICIPATION IN THE STUDY**

Since this is a study that involves a short intervention in the Neonatal Intensive Care Unit, which in past experience has never been associated with negative side effects, your baby will not run specific risks caused by the study itself. However, the knowledge that will be acquired also thanks to his participation may be useful for other newborns.

**WHAT ARE THE BENEFITS THAT YOUR SON / PROTECTED PERSON WILL RECEIVE BY PARTICIPATING IN THE STUDY**

The following benefits are expected from participation in this study: invasive mechanical ventilation is not performed or its duration is reduced during hospitalization with subsequent lower risk of chronic (even potentially serious) respiratory problems in the first months of life and consequent better development neurological; a shorter length of hospital stay.

**RESULTS OF THE STUDY AND CONFIDENTIALITY OF THE INFORMATION COLLECTED**

All the data of your child / your protégé will be pseudo-anonymized, i.e. you will be assigned a code not directly attributable to her person and will be recorded in electronic format. This code will not allow you to identify your child / protected person outside the medical center of the treatment. With regard to the processing of personal data, you must refer to the specific information for the manifestation of consent to the processing of personal data that will be delivered to you at the same time, on a separate sheet.

**WHAT HAPPENS IF YOU DECIDE NOT TO PARTICIPATE IN THE STUDY - POSSIBLE ALTERNATIVE TREATMENTS.**

You are free not to let your child / protege participate in the study. In this case, he / she will continue to receive, however, all the standard therapies and care provided for your pathology, without any penalty, and the doctors will continue to follow you in any case with due care, even if there were no other available therapies (experimental and non-experimental).

**WHAT HAPPENS IN THE EVENT OF DAMAGE**

We inform you that an insurance coverage is active which guarantees any damage to the person deriving from the experimentation according to the indications provided by the …………………………………………………………………………………………..
INFO INSURANCE:…………………………………………

The policy is effective exclusively for damages that occurred no later than …………… from the end of the trial for which a claim has been made within …………months of the end of the same.

However, exceeding the aforementioned ceilings and previous restrictions do not affect your right to request any compensation directly from the person responsible for the damage. By signing this informed consent you are not waiving any of your legal rights.

Before joining the trial in question, if you have signed an insurance policy, you should check with your insurer that your participation has no repercussions on it.

**HOW THEY WILL BE TREATED AND WHO WILL HAVE ACCESS TO THE BIOLOGICAL SAMPLES OF YOUR SON / HIS PROTECTION TAKEN FOR THE PURPOSE OF THE TRIAL**

As for health data, the biological samples of your child / your protégé pseudo-anonymized will also be used for the purposes of the experiment.

Once the experimentation is complete, your child's / protege's samples will be destroyed unless specific informed consent is issued for use for other studies subject to their approval by the Ethics Committee.

**INTERRUPTION OF THE STUDY**

Your participation in this research program is completely voluntary and you can withdraw your child / protegee from the study at any time by notifying the Investigator. In this case, the data collected up to the time of withdrawal will be considered in the results in an aggregate and anonymous form for the final analysis.

Similarly, the trial can be interrupted if:

1. the doctor will not find a benefit or if unwanted effects have occurred, or other;

2. new information became available and the trial was no longer in the best interest of your child / protege;

3. the agreed rules for participation in the trial are not followed;

4. for women (if applicable): your daughter / guardian became pregnant during the trial;

5. the trial was interrupted by the component authorities or by the promoter.

In these cases, you will be promptly informed about further valid treatments for the disease from which your child / protected person is suffering, you will be able to discuss it with the doctor and in any case the center will continue to follow your child / protected person with due care, even in the case there were no other therapies available.

If data becomes available that could influence the decision to continue the study in question, he / she will be promptly informed. If you decide to continue the study, you may be asked to sign a new informed consent in which the update will be highlighted.

**INFORMATION ABOUT THE RESULTS OF THE STUDY**

If you request it, at the end of the study the results of the study in general and in particular those concerning your child / protege may be communicated to you.

**FURTHER INFORMATION**

For further information and communications during the study, you can contact the following staff:

· Prof. …………. – Tel………………….- email……………………..

· Dr. …………. – Tel………………….- email………………………..

· Dr. …………. – Tel………………….- email………………………..

· Dr. …………. – Tel………………….- email………………………..

If you agree, it may be helpful to inform your pediatrician / family doctor that you are participating in this trial, in order to avoid interference with any other medications that you may be prescribed and / or with treatments that you may be undergoing.

The study protocol proposed to you has been prepared in accordance with the Standards of Good Clinical Practice of the European Union and the Declaration of Helsinki of the World Medical Association on clinical trials involving human subjects and has been approved by the Ethics Committee of this facility. you can report any fact you deem appropriate to highlight, in relation to the trial that concerns you, to the Ethics Committee and the Health Department of this structure and to the Competent Authority (AIFA).

**WHO ORGANIZES AND PROMOTES THIS STUDY?**

The study is promoted by the Agostino Gemelli University Hospital Foundation IRCCS.

We thank you for the attention and time you have devoted to reading and discussing this document.

If you decide to involve your child / protege in the Firm, you will be provided with a copy of this Information Sheet and a signed consent form for you to keep.

**DECLARATION OF CONSENT**

(this statement must be signed and dated personally by the Parents or guardian and the doctor who conducted the informed consent discussion)

I, the undersigned: ____________________________ (father)

I, the undersigned ____________________________ (mother)

I, the undersigned ______________________________ (guardian)

from __________________________________________________________________

**I DECLARE**

▢ that I have received from Doctor ________________________________________ exhaustive explanations regarding the request for participation in the research in question, as reported in the information section of which I have been given a copy before now, forming part of this consent, of which I have been delivered a copy on ____________;

▢ that they have been clearly explained to me and I have understood the nature, the purposes, the procedures, the expected benefits, the possible risks and inconveniences and the alternative treatment methods with respect to the proposed clinical trial;

▢ to have had the opportunity to ask any question to the investigator of the study and to have received satisfactory answers;

▢ to have had sufficient time to reflect on the information received; ▢ to have had sufficient time to discuss it with third parties;

▢ to have been informed that the trial protocol and all the modules used have had the favorable opinion of the competent Ethics Committee;

▢ to be aware that the search can be interrupted at any time;

▢ I have been informed that I will be made aware of any new data that could compromise the safety of the research and that, for any problem or further questions, I will be able to contact the principal investigator or his collaborators;

▢ that for the best protection of the health of my child / child I am aware of the importance of informing the pediatrician / general practitioner of the trial in which I agree to involve my child / child.

▢ I am aware of the importance of providing all information (drugs, side effects, etc.) concerning my child / person protected, to the investigator;

▢ I have been informed that the results of the study will be disclosed to the scientific community, protecting the identity of my child / protected according to the current privacy legislation;

▢ to be aware that any choice expressed in this consent form may be revoked at any time and without any justification;

▢ that you have received a copy of this consent form.

Place and date _________________________________________________

Name Surname of parent mother (block letters)
Signature of parent mother

Name Surname of parent parent (block letters)

Signature of parent parent

Name Surname of guardian (block letters)
Signature of guardian

**STATEMENT BY THE DOCTOR WHO GETS THE CONSENT**

I, the undersigned (NAME-SURNAME) ___________________________________ in my capacity as

▢ Principal Investigator

▢ Delegate of the Principal Investigator

**I DECLARE**

That Mr / Mrs (insert name) as parent / guardian has voluntarily consented to participate in the trial.

I also declare that:

▢ have provided the parent / guardian with full explanations regarding the purpose of the trial, the procedures, the possible risks and benefits and its possible alternatives;

▢ have verified that the parent / guardian has sufficiently understood the information provided;

▢ have given the parent / guardian the necessary time and the opportunity to ask questions about the experimentation;

▢ to have clearly illustrated the possibility of having the minor / protected person withdraw from the trial at any time or to modify the choices made;

▢ not having exercised any coercion or undue influence in requesting this consent;

▢ have provided the parent / guardian with information on how the results of the trial will be disclosed to him / her.

_____________________________________________________ ________

Place and date

_________________________________________________ _________________________________________________

Name Surname (block letters) of the doctor who provided the Information and who obtained the consent

Signature (and stamp)

*This form is an integral part and must be kept together with the information form for informed consent*
